# Considerations to practice a safe and minimal administration of heparin and protamine during cardiopulmonary bypass

**DOI:** 10.1051/ject/2026002

**Published:** 2026-06-19

**Authors:** Min-Ho Lee, Kenneth G. Shann, Tami Rosenthal

**Affiliations:** 1 Perfusion Services, Children’s Hospital of Philadelphia Philadelphia PA USA; 2 Division of Cardiac Surgery, Massachusetts General Hospital Boston MA USA

**Keywords:** Target ACT, Protamine-to-heparin ratio, POCT ACT device, Optimal protamine dose, Consumptive coagulation

## Abstract

A critical aspect of cardiopulmonary bypass (CPB) is achieving full anticoagulation to prevent thrombosis and consumptive coagulation (CCA). Systemic anticoagulation with heparin during CPB should be completely neutralized by administering protamine to restore normal hemostasis. Activated clotting time (ACT) is a major marker of anticoagulation management during CPB. However, studies have shown that ACT measurements can vary predictably with different point-of-care testing ACT devices, suggesting the importance of considering the ACT device when determining the target ACT. Otherwise, the amount of heparin used and circulating during CPB can be unnecessarily high. Calculating an optimal protamine dose (PD) is challenging. Yet, newer strategies and heparin concentration-based dosing indicate that average doses of 60–90 mg/m^2^ are generally sufficient. This equates to a protamine-to-heparin ratio of approximately 0.5–0.8:1 of the first heparin bolus to establish the target ACT before going on CPB or a 0.3–0.5:1 of the total heparin dose during CPB. PD exceeding 90 mg/m^2^ may result in residual free protamine. Excessive use of heparin and protamine has been associated with increased post-operative bleeding. This review discusses several considerations in planning an anticoagulation strategy during CPB, focusing on the predictable differences of ACT devices and strategies to determine the optimal PD. The aim is to administer a safe and minimal amount of heparin to protect the CPB circuit and patient from thrombosis and CCA, and to administer the optimal amount of protamine to completely neutralize the circulating heparin without leaving residual free protamine.

## Introduction

A critical aspect of cardiopulmonary bypass (CPB) is achieving systematic anticoagulation to prevent thrombosis, without using excessive amounts of heparin while preventing consumptive coagulation (CCA) by maintaining optimal heparin concentration (HC). It is equally important to restore normal hemostasis post-CPB with an optimal amount of protamine to neutralize circulating heparin completely without residual free protamine. Excessive use of heparin and protamine has been linked to increased post-operative (post-op) bleeding [1–13].

To assure adequate systematic anticoagulation during CPB, activated clotting time (ACT) is monitored using various point-of-care testing (POCT) ACT devices [14–16]. However, there is a significant difference in target ACT (tACT) on CPB among institutions, primarily due to two factors. First, a safe and minimal ACT is not known, and the ideal tACT during CPB is empirical, with variability among institutions, as high as a 20% difference [17–19]. Additionally, maintaining HC of 2.0 international units/mL (IU/mL) or higher, independent from ACT, is recommended to prevent CCA [20–23]. However, HC measurement during CPB is not a common practice, and thus, the anticoagulation management based on HC is not well established. Second, significant differences exist among POCT ACT devices in the long-range ACT. Studies have shown that ACT of the same blood sample can vary significantly with different ACT devices ([16, 24–27]; Table 1). This variation implies that even if two institutions have the same tACT, differences in ACT devices can lead to significant disparities in heparin use and circulating HC during CPB.

**Table 1:** Predictable differences among point of care testing ACT devices.

Reference	Subject (unit)	HMS	HCh SE	ACT Plus	iSTAT	Abrazo
[16]	Avg. bACT (sec)	142	107		124	159
	bACT Ratio (HMS as 1.00)	1.00	0.75		0.87	1.12
	Low ACT reproducibility as *R* value	0.943	0.638		0.946	0.588
	Avg. ppACT (sec)	118	120		113	157
	ppACT Ratio (HMS as 1.00)	1.00	1.02		0.96	1.33
	ppACT/bACT	0.83	1.12		0.91	0.99
	ppACT/bACT Ratio (HMS as 1.00)	1.00	1.35		1.09	1.19
	Avg. CPB ACT (sec)	729	652		600	568
	CPB ACT Ratio (HMS as 1.00)	1.00	0.89		0.82	0.78
	High ACT reproducibility as *R* value	0.947	0.840		0.932	0.868
[25]	Mean ACT difference (sec, HMS as 0)	0	NA		−23	
	All ACT reproducibility as *R* value	NA	0.78		0.97	
[24]	All ACT reproducibility as *R* value		0.780		0.985	
	Mean ACT difference (sec, HCh SE as 0)		0		−20	
[26]	Mean All ACT difference (sec, HCh SE as 0)		0	19.5		
	Mean High ACT difference (sec, HCh SE as 0)		0	34.4		
	Mean ppACT difference (sec, HCh SE as 0)		0	10.3		
[27]	Mean All ACT difference (sec, ACT plus as 0)			0	−87	

At the completion of CPB, the optimal amount of protamine should be administered to restore normal hemostasis from systemic anticoagulation with heparin. An optimal protamine dose (PD) will result in neither residual heparin nor excessive protamine. Protamine under-dosing may cause incomplete heparin neutralization or heparin rebound, leading to increased post-op bleeding [1–4], while excessive PD has also been linked to increased post-op bleeding [5–8], with elevated ACT and decreased platelet function [9–13]. Furthermore, protamine has been shown to have anticoagulant properties in the absence of heparin and other side effects, including anaphylactic response with hypotension, bradycardia, and pulmonary hypertension [7, 28–33]. However, it is also largely empirical how to determine the optimal PD. One common strategy is a fixed protamine-to-heparin ratio (P-to-H) based on either the first heparin bolus (FHB) to establish tACT before going on CPB or total heparin dose (THD) during CPB, which does not account for heparin metabolism. Other strategies have been developed in an effort to consider heparin metabolism and/or HC, such as mathematical calculations of PD using statistical or pharmacokinetic modeling of heparin metabolism over time, or as a function of baseline and post-heparin ACTs, and PD based on a measured HC and calculated total blood volume. Interestingly, nearly all of these new strategies have led to reduced PD, likely decreasing the amount of free protamine after the heparin neutralization [4, 33–43; Table 2].

**Table 2:** Various strategies to determine optimal protamine dose.

Ref	Strategy	ACT device	P:H ratio	Ratio of what	FHB	tACT (sec)	tHC (IU/mL)	Avg PD (mg)	PD (mg/kg)	PD (mg/m^2^)	Per FHB	Per THD
[8]	Fixed ratio	NA	≤0.6:1	THD	300 IU/Kg	450	NA	210	NA	NA	0.70:1*	0.42:1
[8]	Fixed ratio	NA	0.6–1.0:1	THD	300 IU/Kg	450	NA	350	NA	NA	1.17:1*	0.88:1
[8]	Fixed ratio	NA	>1:1	THD	300 IU/Kg	450	NA	500	NA	NA	1.67:1*	1.43:1
[39]	Fixed ratio	HCh SE	0.8:1	THD	300 IU/Kg	450	NA	317*	3.9*	162*	1.05:1*	0.80:1*
[39]	Fixed ratio	HCh SE	0.6:1	THD	300 IU/Kg	450	NA	247*	3.0*	125*	0.82:1*	0.60:1*
[34]	Bull’s curve (control)	M-ACT II	Formula 1	See formula	3 mg/Kg	400	NA	267	NA	148*	0.89:1*	0.63:1
[34]	Mathematical formula using ACT	M-ACT II	1:1	Bull’s curve	3 mg/Kg	400	NA	244	NA	136*	0.81:1*	0.61:1
[40]	Fixed ratio	NA	1:1	FHB	327 IU/kg*	400	NA	280	3.3*	141*	1.0:1	0.78:1*
[40]	PRODOSE Pharmacokinetic model	NA	PRODOSE model	PRODOSE model	330 IU/kg*	400	NA	180	2.2*	94*	0.66:1	0.51:1*
[37]	Fixed ratio	NA	1.1:1	THD	300 IU/Kg	480	NA	416	NA	NA	1.38:1	1.1:1
[37]	Pharmacokinetic model	NA	NA	Pharmacokinetic dosing	300 IU/Kg	480	NA	186	NA	NA	0.62:1	0.50:1
[35]	HC by H-ACT II	H-ACT II	1:1	Circulating heparin amount	388 IU/kg*	480	NA	235	2.9*	121*	0.73:1*	0.51:1*
[35]	HC by H-ACT II	H-ACT II	1:1	Circulating heparin amount	393 IU/kg*	480	NA	246	2.8*	121*	0.72:1*	0.50:1*
[38]	Fixed ratio	HMS	1:1	First heparin bolus + prime	350 IU/kg	480 s	NA	426	5.0*	214*	1.42:1*	NA
[38]	Statistical model	HMS	Statistical Model of HMS	Statistical model of HMS	350 IU/kg	480 s	NA	251	2.9*	125*	0.82:1*	NA
[41]	Fixed ratio	NA	1:1	NA	300 IU/kg	480 s	NA	340	NA	NA	NA	0.60:1*
[41]	HC by HMS	HMS	1:1	Circulating heparin amount	HDR to 550 s target	480 s	HDR value	183	NA	NA	NA	0.42:1*
[42]	HC by HMS	HMS	1:1	Circulating heparin amount	HDR to 550 s target	480 s	HDR value	189	NA	NA	NA	0.59:1
[43]	HC by HMS	HMS	1:1	Circulating heparin amount	352 IU/kg*	480 s	HDR value	210	2.6*	NA	0.80:1	NA
[43]	HC by HMS	HMS	1:1	Circulating heparin amount	361 IU/kg*	480 s	HDR value	170	2.1*	NA	0.64:1	NA
[52]	HC by HMS	HMS	1:1	Circulating heparin amount	307 IU/kg*	400 s	2.0	174	2.1*	88	0.67:1*	0.46:1*
[52]	HC by HMS	HMS	0.9:1	Circulating heparin amount	305 IU/kg*	400 s	2.0	136	1.5*	67	0.50:1*	0.35:1*
[52]	HC by HMS	HMS	0.8:1	Circulating heparin amount	307 IU/kg*	400 s	2.0	121	1.4*	61	0.46:1*	0.32:1*

* Calculated from numbers provided in the manuscript.

M-ACT II; Medtronic ACT II.

H-ACT II; Hepcon ACT II.

A key aspect of anticoagulation management is using a safe and minimal amount of heparin during CPB and administering the optimal amount of protamine at the completion of CPB. While management using ACT only is possible, employing both ACT and HC offers greater accuracy and effectiveness. Unfortunately, only one POCT device commercially available can measure both ACT and HC, the HMS Plus Hemostasis Management System (HMS; Medtronic, Minneapolis, MN). It requires a relatively large volume of blood per assay (~3mL) and provides HC in a stepwise, rather than linear, numbers. Measuring both ACT and HC, along with the heparin dose response test, enables individualized anticoagulant management with an optimal amount of heparin during CPB. HC measurement offers several advantages in anticoagulation management, allowing HC to be maintained above the threshold level to minimize CCA. It also enables calculation of circulating heparin amount at the completion of CPB to administer the optimum amount of protamine [13, 44–54].

## Heparin concentration and consumptive coagulation

Post-op coagulopathy after CPB, even with complete heparin neutralization, is common and likely initiated during CPB. It may result from hemodilution, contact activation of intrinsic factors, and extrinsic factor activation due to surgical procedures. Surgical trauma and CPB components likely increase the inflammatory response [55–58]. Despite systemic anticoagulation with heparin during CPB, thrombin bound to fibrin or clot is not effectively inhibited by heparin-antithrombin III, likely due to a steric hindrance, particularly at the lower concentration of circulating heparin. This can cause continuous activation of the coagulation cascade during CPB and the waste of coagulation factors, leading to CCA [20–23]. These processes can lead to platelet dysfunction, depletion of individual coagulation factors, and hyperfibrinolysis. In addition, post-op coagulopathy is rarely due to incomplete neutralization of circulating heparin, and administering more protamine in response to post-op coagulopathy is unlikely to improve the situation, as excess protamine has an anticoagulant effect by impairing thrombin generation and potentiating fibrinolysis [4–8, 55].

Post-op coagulopathy likely results from multiple factors intrinsic to surgical procedures and CPB itself, which are difficult to control. However, maintaining HC of 2.0 IU/mL or higher, independent of tACT, can minimize CCA. It has been shown that HC of 2.0 IU/mL or higher is required to achieve an effective inhibition of fibrin or clot-bound thrombin, even though it is still partly incomplete [20–23]. Sequestering autologous blood before CPB initiation and retransfusion after CPB can preserve autologous platelets and coagulation factors from CCA [59, 60].

## Significant variability of different POCT ACT devices on tACT and heparin resistance

Even though the anticoagulation strategy during CPB primarily relies on ACT, no gold standard of ACT exists. This lack of standardization has resulted in significant differences among different POCT ACT devices, which likely have clinical implications. In the long-range ACT relevant to CPB, several studies have demonstrated that the same blood sample can yield a predictable difference in ACT. A study by Li et al. [16] showed the predictable difference in long-range ACT in the following order: HMS (100%) > Hemochron Signature Elite (HCh SE) (89%) > iSTAT (82%) > Abrazo (78%). Comparisons between HCh SE and iSTAT (24), as well as HMS and iSTAT (25), further support these findings. Additional studies [26, 27] compared ACT Plus to HCh SE or iSTAT (Table 1). Combining data from these studies reveals the following sequence of the predictable differences: HMS (100%) > ACT Plus (94%) > HCh SE (89%) > iSTAT (82%) > Abrazo (78%) (Table 1). For example, an ACT of 480 s on HMS can be approximately 450 s on ACT Plus, ~430 s on HCh SE, ~395 s on iSTAT, and ~375 s on Abrazo. This suggests that the differences among devices can be clinically significant [16, 24–27].

Institutions using iSTAT or Abrazo may administer significantly more heparin to achieve the same tACT compared to those using HMS or ACT plus, likely resulting in a higher circulating HC. Additionally, institutions using iSTAT or Abrazo may encounter more cases of heparin resistance with the same tACT, potentially leading to excessive heparin administration and unnecessary use of antithrombin III, which may not be necessary for establishing safe anticoagulation for CPB. Higher HCs are associated with an increased risk of heparin rebound and post-op bleeding [2, 5, 17, 18, 61–65].

These facts strongly indicate that the POCT ACT device in use should be considered when determining the tACT (Table 1 and references therein can provide the guidance). Furthermore, when discussing ACT and clinical events related to ACT, such as heparin resistance and protamine dose, it is crucial to specify which POCT ACT device is used to account for predictable differences among the devices.

## ACT and heparin concentration-based anticoagulation strategy

As discussed, the decision of tACT should consider the predictable differences among POCT ACT devices to administer an optimal amount of heparin during CPB [16, 24–27]. Additionally, considering HC would help prevent CCA and excessive heparin administration. Unfortunately, only HMS provides stepwise measurement of HC, leading many institutions to rely solely on ACT for anticoagulation strategy during CPB.

However, HC can be logically estimated, allowing for strategic steps. For example, administering 300 IU/kg at CPB initiation likely results in HC between 4.3 and 5.0 IU/mL in adult women (60–70 mL/kg estimated blood volume) and between 3.8 and 4.3 IU/mL in adult men (70–80 mL/kg estimated blood volume). Heparin is metabolized and diluted during CPB. With a heparin half-life of 90–120 min, HC decreases 2 to 2.5 IU/mL after one half-life, and further decreases may initiate CCA. Administering additional heparin to increase HC can be beneficial even if ACT is higher than tACT at this time. If maintained at a cold temperature, heparin metabolism decreases. Heparin administration can be delayed until rewarming starts if ACT is higher than the target. When rewarming starts, heparin metabolism likely increases, and additional heparin should be administered even if ACT is higher than tACT.

In case of heparin resistance, administering more than 500 IU/kg heparin may not help achieve tACT. The estimated HC would be approximately 7 to 8 IU/mL in adult women and 6 to 7 IU/mL in adult men. When HC is higher than 4 or 5 IU/mL, ACT does not increase further with more heparin administration [66]. Administering more heparin may not achieve tACT and could result in high HC, causing unnecessary post-op bleeding. Using antithrombin III instead of administering any more heparin should be considered.

## Optimal protamine dose

P-to-H should be clearly indicated to fairly compare different studies. The P-to-H of 1:1 typically means administering 1mg of protamine per 100 IU of heparin, with different definitions such as THD administered during CPB, FHB administered to achieve the tACT to go on CPB, or the amount of circulating heparin just prior to the completion of CPB.

A fixed ratio is probably the most commonly employed strategy, with a fixed ratio of either FHB or THD. A fixed ratio of THD is usually somewhere between 0.6:1 and 1:1, while a fixed ratio of FHB is generally 1:1 (Table 2 and references therein). While calculating PD is straightforward, this strategy does not consider heparin metabolism and the actual amount of heparin that needs to be neutralized at the completion of CPB. Several strategies have been developed to administer the optimal amount of protamine, such as a mathematical formula using Bull’s curve, pharmacokinetic models, and statistical modeling. Interestingly, PD was significantly decreased in pharmacokinetic models and statistical modeling studies (Table 2 and references therein).

Another common strategy is based on the amount of circulating heparin right before the completion of CPB, using measured HC and estimated total blood volume. P-to-H in this strategy has generally been 1:1, although a recent study showed that 0.8:1 is sufficient to completely neutralize the circulating heparin without affecting post-op bleeding (Table 2 and references therein).

FHB is usually 300–400 IU/kg to establish tACT in various studies, despite different tACT and POCT ACT devices. However, a significant difference exists in the average PD, ranging from as low as 121 mg to as high as 500 mg in adult institutions (Table 2). A higher PD, and thus inaccurate protamine-heparin matching, likely results in free protamine, which can cause various and sometimes severe side effects and increased post-op bleeding. Even though the half-life of the heparin-protamine complex is known to be short (about 5–8 min) [33, 67], the real half-life of free protamine is not clear. A recent study showed that a significant decrease in post-op bleeding with accurate protamine: heparin matching, starting from 11 hours post-op [43]

Without measuring HC, comparing post-protamine ACT (ppACT) to baseline ACT (bACT) is generally accepted to determine the complete neutralization of heparin. However, one study provided evidence that ppACT/bACT can be significantly different among POCT ACT devices, even though the circulating heparin seems to be completely neutralized [16]. One should consider the ACT device in use when deciding the acceptable ppACT/bACT for complete neutralization of heparin. Once complete neutralization is achieved, no more protamine should be administered, even if there is still oozing. Other options should be explored, such as FFP, platelets, Factor VIIa, and activated prothrombin complex concentrate.

Thromboelastography, with and without heparinase, is another valuable option. It can reveal the presence of residual circulating heparin, coagulopathy, fibrinolysis, or platelet dysfunction and help guide targeted treatment – for example, protamine for complete heparin reversal or FFP/platelets for deficiencies [68–70].

## Summary of strategies

Several factors should be considered when planning an anticoagulation strategy during CPB at each institution. These considerations aim to decrease the amount of heparin and protamine administration while ensuring a safe CPB, minimizing heparin rebound, post-op bleeding, and preventing the adverse effects of protamine overdosing.

First, determining tACT to use a safe and minimal amount of heparin is crucial. Each institution should establish a minimal ACT to maintain as a tACT to protect the CPB circuit and patient from thrombosis. Even if two institutions have the same tACT, the amount of heparin used can vary significantly if different ACT devices are employed. For example, a tACT of 480 s with HMS likely corresponds to approximately 400 s with iSTAT. Consequently, a tACT of 480 s with iSTAT would administer more heparin than the same tACT with HMS and a higher incidence of heparin resistance. In cases of heparin resistance, administering more heparin may be appropriate, but should not exceed 500 IU/kg to avoid an unnecessarily high HC, which could promote non-specific binding to plasma proteins without further increasing ACT, potentially resulting in heparin rebound after CPB [64, 65].

The second consideration is using HC to prevent CCA. While avoiding excessive heparin use is important, maintaining HC of ≥ 2.0 IU/mL, regardless of ACT, is also crucial. CPB gradually inactivates platelets and induces CCA, depleting coagulation factors. Although it may be impossible to entirely prevent CCA, it can be slowed down. Studies have shown that maintaining HC of ≥ 2 IU/mL achieves about 75% inhibition of fibrin-bound thrombin in vitro [20–23].

Third, administering the optimal amount of protamine at the completion of CPB is essential. Studies consistently showed that the optimal PD is critical, and a fixed ratio of 1:1 likely results in protamine overdose. Most new strategies and HC-based dosing indicate that an average dose of 120–180 mg (~60–90 mg/m^2^) is sufficient, corresponding to approximately 0.5–0.8:1 P-to-H of FHB, 0.3–0.5:1 P-to-H of THD, or 0.8–1:1 P-to-H of circulating heparin (Table 2 and references there in). These findings suggest that more than 90 mg/m^2^ is likely to result in free protamine. A recent study by Foubert et al. [71] supports this, showing that an average PD of 289mg or ~147 mg/m^2^ (1:1 P-to-H of circulating heparin by Bull’s curve) led to signs of protamine overdose in 61.1% of patients, while a 60% full dose (~171mg or ~87 mg/m^2^ on average) was sufficient to completely neutralize heparin in 86.5% of patients.

Another consideration is determining the complete neutralization of the circulating heparin. Ideally, direct measurement of HC using HMS is preferred. However, HMS usage is limited, and the comparison of ppACT over bACT is generally employed. The ratio of ppACT/bACT can differ across different POCT ACT devices when circulating heparin is completely neutralized [16]. Thus, if ppACT equal to or less than bACT is a decision point, one ACT device may indicate incomplete heparin reversal, while another device may indicate complete reversal for the same case. This suggests that the ACT device in use should be considered when deciding on complete heparin reversal.

## Conclusion

Excessive use of heparin and protamine has been linked to increased post-op bleeding. This discussion highlights several considerations focusing on the predictable differences of POCT ACT devices and strategies to determine the optimal PD. When planning the anticoagulation strategy during CPB, the goal should be administering a safe and minimal amount of heparin to protect the CPB circuit and patient from thrombosis while maintaining HC of ≥ 2.0 IU/mL to slow down CCA, and to administer the optimal amount of protamine to completely neutralize heparin without residual free protamine.

## A glossary of abbreviations

ACT: activated clotting time
bACT: baseline ACT
CCA: consumptive coagulation
CPB: cardiopulmonary bypass
FHB: first heparin bolus
HC: heparin concentration
HCh SE: Hemochron Signature Elite
HMS: HMS Plus Hemostasis Management System
IU/mL: international units/mL
PD: protamine dose
POCT: point-of-care testing
Post-op: post-operative
ppACT: post-protamine ACT
P-to-H: protamine-to-heparin ratio
tACT: target ACT
THD: total heparin dose

## Data Availability

This was a literature review. Thus, no patient data was collected.
